# (Pro)renin receptor antagonist PRO20 attenuates nephrectomy‐induced nephropathy in rats via inhibition of intrarenal RAS and Wnt/β‐catenin signaling

**DOI:** 10.14814/phy2.14881

**Published:** 2021-05-31

**Authors:** Yan Wang, Yurong Wang, Kai Xue, Huaijie Wang, Jingjing Zhou, Feng Gao, Chengde Li, Tianxin Yang, Hui Fang

**Affiliations:** ^1^ Key Laboratory of Applied Pharmacology in Universities of Shandong Department of Pharmacology School of Pharmacy Weifang Medical University Weifang China; ^2^ Department of Internal Medicine University of Utah and Veterans Affairs Medical Center Salt Lake City UT USA

**Keywords:** 5/6 nephrectomy, (Pro)renin receptor, renin‐angiotensin system

## Abstract

**Introduction:**

(Pro)renin receptor has emerged as a new member of the renin‐angiotensin system implicated in the pathogenesis of chronic kidney disease (CKD). Herein we report characterization of the therapeutic potential of (pro)renin receptor (PRR) antagonist PRO20 in 5/6 nephrectomy (5/6Nx) rats.

**Methods:**

Male Wistar rats underwent 5/6Nx followed by treatment with vehicle or received daily injections of a PRR inhibitor PRO20 (700 μg/kg) via the 3 s.c. Sham group served as a control.

**Results:**

As compared with the sham control, the 5/6Nx rats exhibited significant increases in proteinuria, glomerulosclerosis, tubular injury, and interstitial inflammation in the remnant kidneys. Treatment with PRO20 significantly attenuated these abnormalities, as evidenced by reduced expression of fibronectin, α‐SMA, collagen 1, TGF‐β1, IL‐6, IL‐8, IL‐1β, MCP‐1 and increased expression of E‐cadherin. Increased urinary/renal levels of renin activity, angiotensinogen (AGT), and Angiotensin II (Ang II) by 5/6Nx, which were all ameliorated by PRO20. Renal PRR, the secreted proteolytic fragment of PRR (sPRR) in renal and urinary, were all elevated in 5/6Nx rats. Moreover, our results revealed that renal Wnt3A and β‐catenin expression were upregulated during 5/6Nx, which were all attenuated by PRO20.

**Conclusions:**

Overall we conclude that in vivo antagonism of PRR with PRO20 will improve 5/6Nx‐induced CKD mainly through inhibition of intrarenal RAS and Wnt/β‐catenin signaling pathway.

## 1 INTRODUCTION

The renin‐angiotensin system (RAS) is undisputed regulator in the development of cardiovascular, hypertensive, and renal diseases (Li et al., 2017). Apart from systemic RAS, the intrarenal RAS plays a key role in the pathophysiology of chronic kidney diseases (CKD) (Yang & Xu, 2017). Therefore, inhibition of the intrarenal RAS is crucial in restricting the development of CKD.

(Pro)renin receptor (PRR) is a novel member of the RAS and has been implicated in regulation of intrarenal RAS during hypertension and CKD(Nguyen et al., 2002). PRR can bind prorenin/renin and induce multiple pathways, including fibrotic response (Clavreul et al., 2011; Huang et al., 2006), oxidative stress (Peng et al., 2013), Wnt/β‐catenin signaling (Cruciat et al., 2010), mitogen‐activated protein kinase (MAPK) (Saris et al., 2006) via RAS‐dependent and RAS‐independent manners.

The handle region peptide (HRP) was originally used as an inhibitor of PRR but experimental results with HRP are somewhat controversial (Ichihara et al., 2004). For example, HRP can alleviate organ damage and eyeball diseases caused by diabetes and hypertension, but it does not reduce the hypertension of renin and angiotensinogen (AGT) overexpressing rats (Feldt et al., 2008; Giese & Speth, 2014; Seki et al., 2010). A more problematic fact is that the HRP does not block PRR‐mediated activation of ERK1/2 phosphorylation in vascular smooth muscle cells, a result thought to strongly challenge HRP as a PRR inhibitor (Feldt et al., 2008). PRO20 is a second version of PRR decoy inhibitor after HRP (Li et al., 2015). It is a 20‐amino‐acid peptide (sequence of first 20 amino acid residue segments of prorenin prosegment: L^1^PTDTASFGRILLKKMPSVR^20^), containing all the sites where PRR binds to renin or (pro)renin. PRO20 and HRP both compete to bind with the handle region of the renin or (pro)renin, which prevents the PRR from binding to it, thereby inhibiting the PRR‐induced renin activity. PRO20 is composed of 20 amino acids, which is far longer than the 10 amino acid HRP. PRO20, as a specific antagonist of PRR, has been confirmed by more recent studies (Fang et al., 2018; Lu et al., 2016; Peng et al., 2017; Su et al., 2017; Wang et al., 2015, 2016; Xu et al., 2016; Xu, Lu, Lu, et al., 2017; Xu, Lu, Wang, et al., 2017). Recent research also suggests that PRR can promote renal injury by enhancing Wnt/β‐catenin signaling (Li, Zhou, et al., 2017) and activation of RAS (Xiao et al., 2019).

In sum, these data suggest that targeting PRR may be better protected against CKD. So far, no prior study examines the therapeutic potential of PRO20 in 5/6Nx rats, a well‐defined model of CKD.

## 2 MATERIALS AND METHODS

### 2.1 Animals

Male Wistar rats (6 weeks old, 160 to 180 g) were purchased from Ji'nan Pengyue Laboratory Animal Breeding Co., Ltd. All animals were housed in temperature‐controlled cages (five animals/cage) with a 12‐hour light‐dark cycle and 25°C. The animal protocol was approved by Institutional Animal Care and Use Committee of Weifang Medical University, China.

### 2.2 Animal modeling

Rats were acclimated for 1 week before the start of experiments. They were given with 10% chloral hydrate (3.0 ml/kg, i.p.) anesthesia and then underwent sham operation or 5/6Nx. The 5/6Nx model was prepared according to the two‐step protocol (Figure 5d) as described previously (Li et al., 2007). Briefly, 5/6 Nx rats underwent surgical resection of the upper and lower thirds of the left kidney and right nephrectomy after one week. The sham‐operated animals were used as controls. The excised sections of the left and right kidneys were weighed immediately after removal.

### 2.3 Drug administration and grouping

One week after the surgery, the success of the modeling was that the serum creatinine of the modeling group was significantly higher than that of the control group (data not shown). The rats were divided into three groups of six: sham control group (CTR group), 5/6Nx group, and PRO20 treatment group (5/6Nx + PRO20 group). The rats of 5/6Nx + PRO20 group were administrated with PRO20 (700 μg/kg/d via subcutaneous injection for mean three doses every single day for 14 weeks).

### 2.4 Sample collection and preparation

After 12 weeks of treatment, rats were placed inside individual metabolic cages for 24 h and urine was collected, and the rats were weighed and subsequently sacrificed with chloral hydrate (10%, 3 ml/kg i.p.) followed by blood samples were collected from abdominal vena cava. The left kidneys were removed, weighted, and cut into several parts. Some renal tissues were frozen in liquid nitrogen and stored for protein and mRNA analysis; others were fixed for morphological studies.

### 2.5 Changes in renal function and proteinuria

The blood urea nitrogen (BUN), serum creatinine (Scr), and proteinuria were determined by using commercial kits (Nanjing Jiancheng Bioengineering Institute).

### 2.6 Measurement of systolic blood pressure (SBP)

Rat SBP was determined using the noninvasive small animal tail blood pressure system (Apex) as previously described (Jia et al., 2012). All animals were habituated to the test environment for 1 week before measurement. The mean SBP of each animal was measured three times.

### 2.7 Histopathologic assessment

Formalin‐fixed paraffin‐embedded one‐fourth of the kidneys were sectioned to 5 μm thickness. Kidney histology was examined after Periodic Acid Schiff (PAS) staining. Glomerular damage was evaluated by two independent and experienced observers. Glomerular damage was assessed with a semi‐quantitative scoring system, as previously described (Raij et al., 1984; Wang et al., 2017; Zhu et al., 2011). In brief, a minimum of 20 glomeruli in each specimen were examined and the severity of lesions were graded from 0 to 4 according to the percentage of glomerular involvement. Thus, 0, no changes; 1 = less than 25% of glomerular area involved; 2 = between 25 and 50% of glomerular area involved; 3 = between 50 and 75% of glomerular area involved; and 4 = more than 75% of tuft area involved. The average scores from counted glomeruli were used as the glomerular damage index for each animal. Tubulointerstitial indexes were assessed with a semi‐quantitative scoring system, as previously described (Gadola et al., 2004; Veniant et al., 1994). Briefly, the severity of tubulointerstitial injury was scored by two independent observers according to the percentage of damages including massive infiltration of inflammatory cells, tubular atrophy and dilation, protein casts (the higher the score was, the more severe the injury): 0, no changes; 1, lesions involving <25% of the cortical area; 2, lesions between 25% and 50% of the cortical area; and 3, lesions involving >50% of the cortical area. The index in each group was expressed as the mean of all scores obtained. Images were captured under a Leica microscope (Leica Microsystems). Ten random pictures per animal were quantified.

### 2.8 Immunostaining

Formalin‐fixed, paraffin‐embedded specimens (5 μm) from the samples were used for immunohistochemical analysis. The slides were incubated with 0.3% H_2_O_2_ in absolute methanol for 30 min to block endogenous peroxidase activity. The slides were then blocked with phosphate‐buffered saline (PBS)/0.5% bovine serum albumin (BSA) for 30 min at room temperature and overnight incubation with primary antibody against PRR (rabbit polyclonal immunoglobulin G (IgG); Abcam) at 4℃. This was followed by incubation with a biotinylated goat antirabbit IgG diluted 1: 200 in PBS as a secondary antibody (Boster Biological Technology Co., Ltd) in a humidified box at room temperature for 60 min. The slides were then incubated with 50 ml of diaminobenzadine (Boster Biological Technology Co., Ltd) as a substrate, and counterstained with 10% Mayer's hematoxylin (Leagene) and cover‐slipped with Permount (HAORAN Biological Technology Co., Ltd), mounted and observed with a Leica fluorescence microscope (Leica Microsystems). A semi‐quantitative method was used to evaluate the percentage of positive staining area in the glomeruli. Images were analyzed with Image‐Pro Plus 4.5 software (Media Cybernetics, Inc.). The brown areas were judged as positive.

### 2.9 Immunofluorescence

Formalin‐fixed, paraffin‐embedded specimens (5 μm) from the samples were used for Immunofluorescence analysis. The slides were labeled with antibodies to active‐β‐catenin (1:100, 05‐665; EMD Millipore).

The secondary antibodies were Alexa Fluor 594‐conjugated goat anti‐mouse IgG (1:1000, Invitrogen). Nuclei were counterstained with 4′6‐diamidino‐2‐phenylindole (DAPI, Sigma‐Aldrich). After PBS washing, the slides were mounted with aqueous mounting medium (CTS011, BD Bioscience) and cover‐slipped.

### 2.10 Western immunoblotting

Western immunoblotting was carried out as previously described (Wang et al., 2014). In short, thirty micrograms of protein for each sample were denatured in metal bath for 10 min, separated by SDS‐PAGE, and transferred onto polyvinylidene ﬂuoride (PVDF) membranes (Immobilion‐P, Millipore). Membranes were blocked for 1 h with Tris‐buffered saline with Tween‐20 (TBST) containing 5% nonfat dry milk at room temperature, followed by incubation with indicated primary antibodies at 4°C overnight (PRR, 1:1000 dilution, HPA003156, Sigma‐Aldrich; Santa Cruz; fibronectin (FN), 1:1000 dilution, F3648, Sigma‐Aldrich; Collagen 1 (COL‐1), 1:1000 dilution, sc‐59772, Santa Cruz; α‐smooth muscle actin (α‐SMA), 1:1000 dilution, A5228, Sigma‐Aldrich; E‐cadherin, 1:1000 dilution, SAB4503751, Sigma‐Aldrich; interleukin 8 (IL‐8), 1:1000 dilution, sc‐8427, Santa Cruz; transforming growth factor β1 (TGF‐β1), 1:1000 dilution, ab31013, Abcam; Wnt3A, 1:1000 dilution, 09‐162, EMD Millipore; Active‐β‐Catenin, 1:1000 dilution, 05‐665; EMD Millipore; β‐actin, 1:10000 dilution, AA132, Beyotime Biotech Inc.) for overnight at 4°C. Bound antibodies were visualized by using enhanced chemiluminescence technology. Blots were quantified by densitometry using Fluor Chem FC3 image analyzer (Molecular Devices, USA). β‐Actin served as the internal reference.

### 2.11 ELISA assays for renin, soluble PRR (sPRR), AGT and Ang II

Renin activity activity assay was performed as described previously (Campbell et al., 2009). In short, plasma and urine samples were centrifuged at 4000 rpm for 20 min at 4°C and the supernatants were subsequently collected. Renin activity was determined by the generation of delta value of the Ang I generation after 1‐h incubation at 37°C versus 4°C using commercial ELISA kits. The concentration of urine and plasma Ang I was measured using an angiotensin I EIA kit (S‐1188, Peninsula laboratories international) according to the manufacturer's instruction manual. Plasma and urine sPRR, AGT and Ang II were measured using the commercial available ELISA kit (27781, Immuno‐Biological Laboratories, Takasaki, Japan; SEA797Ra; CEA005Ra, *Cloud‐Clone Corp*.).

### 2.12 Measurement of thiobarbituric acid reactive substances (TBARS)

Malondialdehyde (MDA) formation was used as an indicator of TBARS production by employing a commercially available kit (10009055, Cayman Chemical) (Kobori et al., 2007; Wang et al., 2010).

### 2.13 Quantitative reverse transcriptase PCR (qRT‐PCR)

Snap frozen renal samples were homogenized in TRIzol® Reagent (CWBio). Total RNA isolation/purification and reverse transcription reactions were done as described (Paliege et al., 2004). RNA samples were treated with RNAse free DNase kit (Kirgen) prior to reverse transcription. RNA was converted into cDNA using Reverse Transcription Kit (Toyobor). Primers were designed and provided by Shanghai Sangon Company (Table 1). qRT‐PCR was conducted using SYBR Green Master Mix (Toyobo) on a Light Cycler 480 Real‐Time PCR System (Roche). The PCR programs: 95°C 10 min, 40 cycles for 95°C 5 s, 55°C 10 s and 72°C 15 s, followed by an extension at 72°C for 7 min. The data was shown as a relative value normalized by GAPDH.

**TABLE 1:** Primer sequences designed for qRT‐PCR

Target gene	Primer sequence
Rat GAPDH F	AGACAGCCGCATCTTCTTGT
Rat GAPDH R	TTCCCATTCTCAGCCTTGAC
Rat FN F	AGACCATACCTGCCGAATGTAG
Rat FN R	GAGAGCTTCCTGTCCTGTAGAG
Rat α‐SMA F	GGAGCATCCGACCTTGCTAA
Rat α‐SMA R	CCATCTCCAGAGTCCAGCAC
Rat COL‐1 F	ACGCATGGCCAAGAAGACATCCC
Rat COL‐1 R	TTGCATTGCACGTCATCGCACAC
Rat E‐cadherin F	ACAAAGACAAAGAAGGCAAGGTTT
Rat E‐cadherin R	AGAGTGTATGTGGCAATGCGTT
Rat IL‐1β F	GAGCTGAAAGCTCTCCACCT
Rat IL‐1β R	TTCCATCTTCTTCTTTGGGT
Rat IL‐6 F	GCCAGAGTCATTCAGAGCAATA
Rat IL‐6 R	GTTGGATGGTCTTGGTCCTTAG
Rat IL‐8 F	CCCCCATGGTTCAGAAGATTG
Rat IL‐8 R	TTGTCAGAAGCCAGCGTTCAC
Rat MCP‐1 F	TAGCATCCACGTGCTGTCTC
Rat MCP‐1 R	CAGCCGACTCATTGGGATCA
Rat TGF‐β1 F	CTCAACACCTGCACAGCTCC
Rat TGF‐β1 R	AGTTGGCATGGTAGCCCTTG
Rat PAI‐1 F	TGGTGAACGCCCTCTATTTC
Rat PAI‐1 R	GAGGGGCACATCTTTTTCAA
Rat Nephrin F	CTGACCACACCAACATCCAG
Rat Nephrin R	AGGCAGACCCCCATCAAAG
Rat Wnt3A F	ATGGGCGGGAGGGGAGAGAT
Rat Wnt3A R	CGCCCCCATTGGATCCTTAAG
Rat β‐catenin F	CTGACCACACCAACATCCAG
Rat β‐catenin R	AGGCAGACCCCCATCAAAG
Rat snail F	TGCACGACCTGCGAAAG
Rat snail R	TGTGGAGCAAGGACATTCG
Rat MMP‐7 F	TCTAGGCCATGCCTTTGCA
Rat MMP‐7 R	TCCGTCCAGTACTCATCCTTGTC
Rat Fsp‐1 F	ACCTCTCTGTTCAGCACTTCC
Rat Fsp‐1 R	GAACTTGTCACCCTCGTTGC
Rat PRR F	ATCCTTGAGACGAAACAAGA
Rat PRR R	AGCCAGTCATAATCCACAGT

### 2.14 Statistical analysis

Data was analyzed by Graphpad prism 6 software. Data are summarized as means ± SEM. Statistical analysis was performed by using analysis of variance with the Bonferroni test for multiple comparisons, or by unpaired student t‐test for two comparisons. A *p* value less than 0.05 was considered statistically significant.

## 3 RESULTS

### 3.1 Using PRO20 could attenuate proteinuria and improved renal damage in 5/6Nx rats

The left and right weight of removed kidneys were no different between 5/6Nx and 5/6Nx + PRO20 groups (2.01 ± 0.01 vs. 2.00 ± 0.08, *p* > 0.05). 5/6Nx rats displayed polyuria, which was nearly completely reversed by PRO20 treatment; renal function was indicated by the levels of BUN and Scr, which were tested to determine the effects of PRO20 on renal function at 12 weeks (at the end of intervention). The 5/6Nx rats showed significantly increased level of BUN and Scr, as compared with the CTR rats as shown in Table 2 (*p < *0.05). Moreover, the concentrations of BUN, Scr, and 24 h proteinuria in rats treated with PRO20 were significantly depressed as compared to the 5/6Nx rats (*p* < 0.05). Table 2 shows the SBP value was elevated in the 5/6Nx group as compared with the sham control, and PRO20 alleviated the elevation in BP induced by 5/6Nx at the end of the experiment. Thus, these results support renoprotective and antihypertensive actions of PRO20 in the 5/6Nx model.

**TABLE 2:** Effect of PRO20 on biochemical and renal injury biomarkers in the experimental rats

Group	CTR	5/6Nx	5/6Nx + PRO20
SBP (mm Hg)	122 ± 1.33	143 ± 2.73*	128 ± 4.70^#^
Final Body Weight (g)	444 ± 9.58	326 ± 11.62*	367 ± 12.94* ^,^ ^#^
Weight Gain (g)	167 ± 8.65	89 ± 4.67*	124 ± 10.07* ^,^ ^#^
Water Intake(ml/24 h)	33.41 ± 1.02	106.43 ± 5.80*	78.69±6.07* ^,^ ^#^
Urine Volume(ml/24 h)	11.9 ± 0.21	70.5 ± 10.33*	42.7 ± 4.37* ^,^ ^#^
Urinary protein excretion(mg/24 h)	18.49 ± 0.26	259.58 ± 29.46*	129.16 ± 29.03* ^,^ ^#^
Plasma creatinine(μmol/L)	128.90 ± 2.47	342.88 ± 31.14*	138.94 ± 3.15* ^,^ ^#^
Plasma urea nitrogen(mmol/L)	10.96 ± 0.08	47.43 ± 1.81*	30.85 ± 0.73* ^,^ ^#^

Data are means ± SEM.

N = 6.

SBP, systolic blood pressure.

* *p* < 0.05 versus CTR.

# *p* < 0.05 versus 5/6Nx.

**FIGURE 1:**
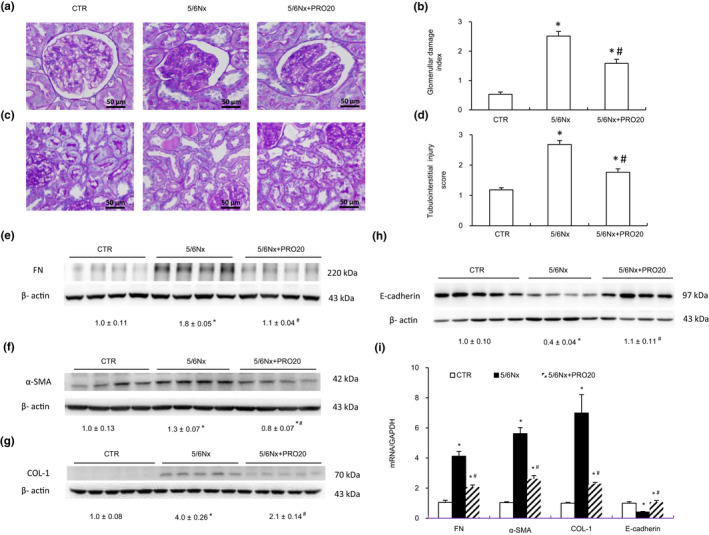
Histological analysis of renal injury. Male Wistar rats were treated for 12 weeks with control, 5/6Nx, or 5/6Nx + PRO20. (a) Representative photomicrographs showing glomerular structures (periodic acid‐schiff staining, 400×) and (b) summarized glomerular damage index by semiquantitation of scores in different groups. (c) PAS staining of renal cortex. Original magnification, ×200. (d) Renal tubulointerstitial injury score from semi‐quantitative analysis of renal pathologies. Average of 10 fields of area per rat. N = 5 per group. (e, f, g, and h) Immunoblotting analysis of FN, α‐SMA, COL‐1, E‐cadherin. The protein abundance of FN, α‐SMA, COL‐1, E‐cadherin protein levels calculated by densitometry were normalized relative to β‐actin signals. (i) qRT‐PCR analysis of mRNA expression of FN, α‐SMA, COL‐1, E‐cadherin in renal cortex. The expression values were normalized to that of GAPDH. N = 6 per group. *p < 0.05 versus CTR; ^#^ *p* < 0.05 versus 5/6Nx

By PAS staining analysis, the 5/6Nx group developed glomerular sclerotic damages as indicated by occlusion of glomerular capillaries and mesangial matrix expansion (Figure 1a). The glomerular damage index was substantially higher in 5/6Nx rats; in contrast, 5/6Nx‐induced glomerular damage was significantly attenuated by PRO20 (Figure 1b). Additionally, the 5/6Nx group exhibited interstitial inflammatory cell infiltration, protein casts, partial tubular expansion, and severe tubular atrophy cells were observed (Figure 1c). PRO20 improved the histological damage induced by 5/6Nx (Figure 1c). These results were semi‐quantitatively analyzed by showing tubulointerstitial injury score (Figure 1d).

Furthermore, the protein and mRNA expression of FN, α‐SMA, COL‐1 was detected using western blot and qRT‐PCR. Western blot analysis demonstrated a reduced protein expression of FN, α‐SMA, COL‐1 in remnant kidney treated with PRO20 (Figure 1e–g). qRT‐PCR indicated that PRO20 treatment could decrease the mRNA levels of FN, α‐SMA, COL‐1 induced by 5/6Nx (Figure 1i). E‐cadherin protein expression was significantly decreased after 5/6Nx, and PRO20 treatment restored the expression of E‐cadherin at both mRNA and protein levels (Figure 1h,i).

In addition, we examined expression of nephrin, a marker of podocyte injury. By qRT‐PCR, renal cortical nephrin mRNA levels was significantly decreased in 5/6Nx as compared with sham control (0.66 ± 0.04 in 5/6Nx group vs. 1.00 ± 0.01 in CTR group, N = 5, *p*<0.05). The nephrin level was restored by PRO20 (0.66 ± 0.04 in 5/6Nx vs. 1.08 ± 0.16 in 5/6Nx + PRO20, *p* < 0.05).

### 3.2 Using PRO20 could attenuate renal inflammation, fibrosis and oxidative stress in 5/6Nx rats

**FIGURE 2:**
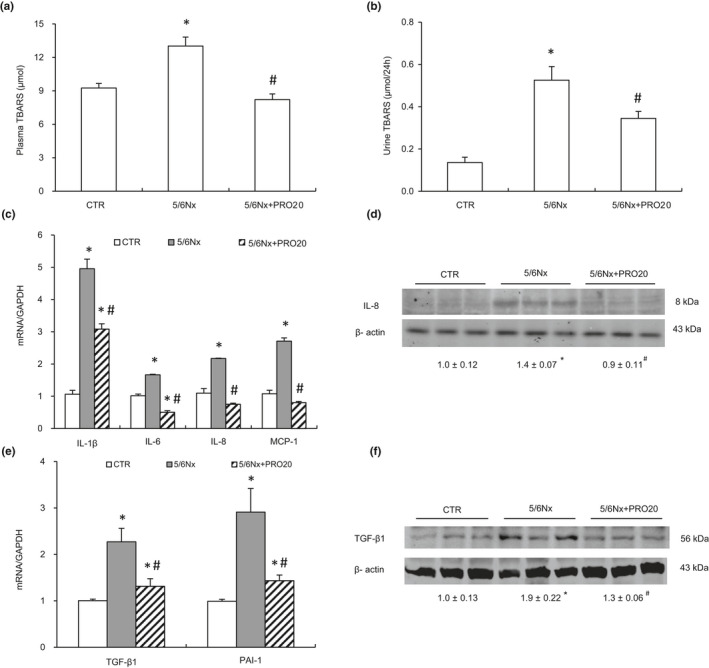
Analysis of the expression levels of renal oxidative stress and pro‐inflammatory cytokines. (a and B) Plasma and urinary TBARS. (C) qRT‐PCR analysis of mRNA expression of IL‐1β, IL‐6, IL‐8 and MCP‐1 in renal cortex. The values were normalized by GAPDH. (d) Immunoblotting analysis of IL‐8. The protein abundance of IL‐8 was analyzed by densitometry and the values were normalized by β‐actin and shown underneath the blot. (e) qRT‐PCR analysis of mRNA expression of TGF‐β1, PAI‐1 in renal cortex. The expression values were normalized to that of GAPDH. (f) Immunoblotting analysis of TGF‐β1. The densitometry of TGF‐β1 was normalized by β‐actin. Data are means ± SEM. N = 6 per group. *p < 0.05 versus CTR; ^#^ *p* < 0.05 versus 5/6Nx

CKD is featured by oxidative stress and inflammation; which are inseparably linked and play a key role in driving the development and progression of CKD as well as other complications (Ruiz et al., 2013). Thus, inhibiting oxidative stress may delay the progression of CKD. The plasma and urine levels of TBARS were examined as a marker of oxidative stress. Plasma and urine TBARS presented a significant increase in the 5/6Nx group as compared with the CTR group and this elevation was less in the 5/6Nx + PRO20 group (*p* < 0.05, Figure 2a,b).

Given the well‐reocngized role of inflammation in CKD, we assessed expression of various proinflammatory factors such as interleukin 1β (IL‐1β), interleukin 6 (IL‐6), interleukin 8 (IL‐8) and chemoattractant protein 1 (MCP‐1) using qRT‐PCR analysis. As shown in Figure 2c, mRNA levels of IL‐1β, IL‐6, IL‐8 and MCP‐1 was significantly increased in 5/6Nx rats and these increases were less in the 5/6Nx + PRO20 rats (*p* < 0.05, Figure 2c). Subsequently, the selected cytokines such as IL‐8 expression was validated using western blot analysis.

The expression of renal IL‐8 protein was significantly increased in the 5/6Nx rats as compared with the CTR group (*p* < 0.05), which was blocked by PRO20 (*p* < 0.05, Figure 2d).

Fibrosis is the excessive accumulation of extracellular matrix components, and the accumulated fibrotic tissues disarrange the renal constitution. Renal fibrosis is the common final pathway of renal damage in CKD, regardless of its etiology. We finally evaluated various fibrosis‐related markers. To investigate the effects of PRO20 on renal fibrosis in 5/6Nx rats, we measured the mRNA and protein expression of renal fibrosis regulators (TGF‐β1) by qPCR and western blotting, respectively. As shown in Figure 2e,f, PRO20 significantly suppressed mRNA levels of TGF‐β1 (*p* < 0.05, Figure 2e) as well as its protein expression (*p* < 0.05, Figure 2f). In addition, PRO20 also significantly suppressed mRNA levels of plasminogen activator inhibitor‐1 (PAI‐1) (*p* < 0.05, Figure 2e). In sum, these results indicated that PRO20 may have a renoprotective role in 5/6Nx rats.

### 3.3 Using PRO20 could inhibit intrarenal RAS activation induced by 5/6Nx

**FIGURE 3:**
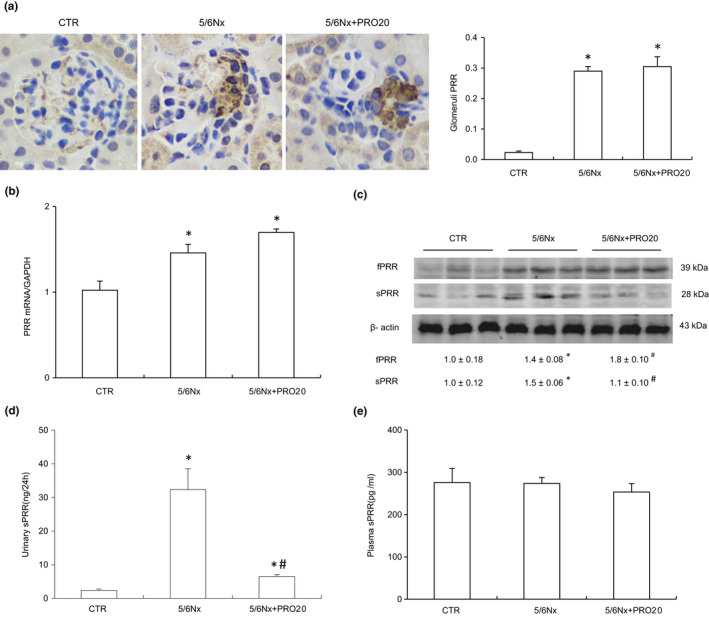
Analysis of PRR/sPRR expression. (a) Representative DAB IHC‐stained sections of the glomeruli demonstrating the expression of PRR. DAB, diaminobenzidine, IHC, immunohistochemistry, original magnification ×400. (B) qRT‐PCR analysis of renal cortex PRR expression. The expression values were normalized to that of β‐actin. (c) Renal expression of full‐length PRR and sPRR by immunoblot analysis. The data shown is a representative image. The expression values were normalized to that of β‐actin. (d) ELISA analysis of urinary sPRR excretion. (e) ELISA analysis of plasma sPRR. Data are means ± SEM. N = 6 per group. *p < 0.05 versus CTR; ^#^ *p* < 0.05 versus 5/6Nx

Inappropriate activation of the intrarenal RAS was a critical factor to the pathogenesis of 5/6Nx‐induced nephropathy as well as other types of CKD (Bian et al., 2019; Ishigaki et al., 2018; Luo et al., 2020). The RAS has been demonstrated to contribute to the progression of CKD (Urushihara & Kagami, 2017). In addition, PRR has recognized as a key component of the intrarenal RAS during albumin overload‐induced nephropathy (Fang et al., 2018). Immunohistochemical staining indicated the localization of PRR expression predominantly in the glomeruli (Luo et al., 2020). The intensity and area of PRR staining were significantly increased in the glomeruli of 5/6Nx rats as compared with the CTR group, which was unaffected by PRO20 treatment (Figure 3a).

**FIGURE 4:**
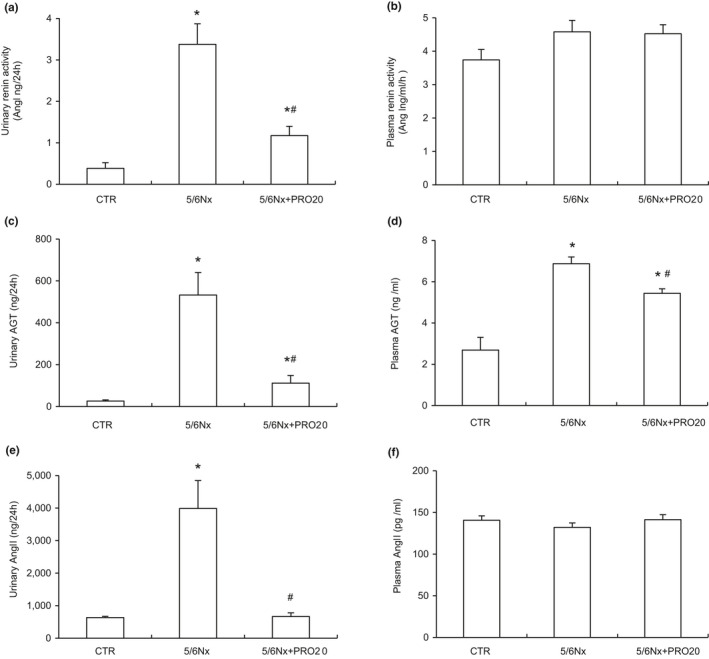
Analysis of intrarenal renin levels in comparison with plasma renin activity. (a) Urinary renin activity. (b) Plasma renin activity. (c) ELISA analysis of urinary AGT. (d) ELISA analysis of plasma AGT. (e) ELISA analysis of urinary Ang II. (f) ELISA analysis of plasma Ang II. Data are means ± SEM. N = 6. *p < 0.05 versus CTR; ^#^ *p* < 0.05 versus 5/6Nx

We further investigated the effects of PRR may regulate the activity of intrarenal RAS in 5/6Nx rats. The 5/6Nx rat model showed elevated renal PRR mRNA and protein expression and urinary sPRR secretion compared to CTR group (*p* < 0.05, Figure 3b–d), contrasting to unchanged plasma sPRR (Figure 3e). Taken together, these results provide compelling evidence that activation of intrarenal PRR/sPRR. Similarly, urinary renin activity was significantly increased in the 5/6Nx group (*p* < 0.05, Figure 4a). However, circulating renin activity remained on a similar constant level (Figure 4b). PRO20 dramatically attenuated 5/6Nx ‐induced urinary renin activity (3.38 ± 0.49 in 5/6Nx vs. 1.17 ± 0.22 Ang I ng /24 h in 5/6Nx + PRO20, *p* < 0.05, Figure 4a). Furthermore, urinary and plasma AGT and Ang II levels were significantly increased as compared with CTR group (*p* < 0.05), which were both suppressed by PRO20 treatment (*p* < 0.05, Figure 4c–e). Conversely, Ang II levels in plasma concentrations were similar among the three groups (Figure 4f). These data suggest that 5/6Nx‐induced intrarenal RAS activation is PRR dependent but not systemic RAS.

### 3.4 Using PRO20 could inhibit Wnt/β‐catenin signaling activation in 5/6Nx rats

**FIGURE 5:**
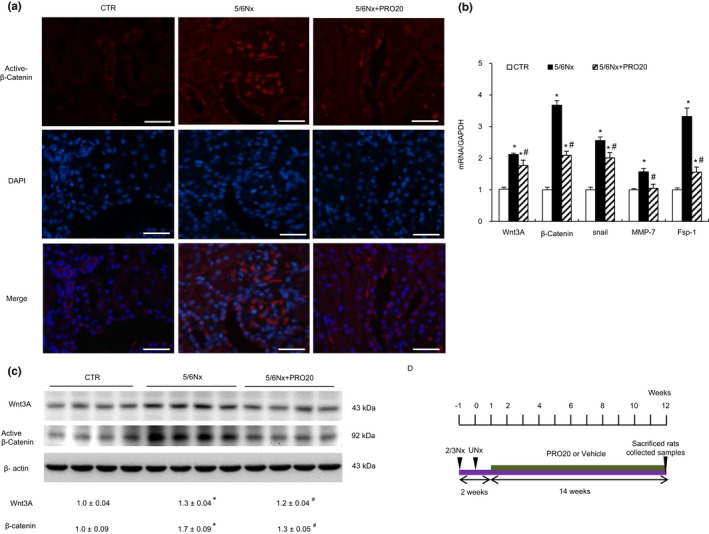
(a) Immunofluorescence microscopy show Nx‐induced β‐catenin activation that was attenuated by PRO20. Scale bars indicate 50 μm. (b) qRT‐PCR analysis of mRNA expression of Wnt3A, β‐catenin, snail, MMP‐7, Fsp‐1 in renal cortex. The expression values were normalized to that of GAPDH. (c) Immunoblotting analysis of Wnt3A, β‐catenin in renal cortex. The data shown is a representative image. The expression values were normalized to that of β‐actin. (d) Diagram shows experimental design. Green bar shows the treatment schedule of PRO20 or vehicle. N = 6. *p < 0.05 versus CTR; ^#^ *p* < 0.05 versus 5/6Nx

Activation of Wnt/β‐catenin signaling is a common pathologic finding in a wide variety of CKD, regardless of their etiologies (He et al., 2009). Recent studies indicate that PRR acts as an essential component of the Wnt/β‐catenin signaling and is obligatory for its signal transduction (Li, Zhou, et al., 2017). We next investigated the PRR regulation of Wnt/β‐catenin signaling in 5/6Nx. As shown in Figure 5a, immunofluorescence exhibited that β‐catenin protein was induced in remnant kidney at 12 weeks after 5/6Nx, as compared with CTR. Immunofluorescence staining also showed that PRO20 reduced the β‐catenin expression. The mRNA levels of Wnt3A, β‐catenin, snail, matrilysin (MMP‐7), fibroblast‐specific protein‐1 (FSP‐1) were considerably upregulated at 12 weeks after 5/6Nx, compared with CTR, which was inhibited by PRO20 (Figure 5b). Western blot analyses also revealed that 5/6Nx induced Wnt3A and active‐β‐catenin proteins (Figure 5c). Interestingly, PRO20 did not significantly affect Wnt3A induction by Nx in rat cortex (Figure 5c), but it primarily blocks active‐β‐catenin. These results demonstrate that PRO20 has an attenuating effect on Wnt/β‐catenin signaling activation in 5/6Nx.

## 4 DISCUSSION

The present study for the first time demonstrates that PRO20 treatment exerts renoprotective action in a rat model of 5/6Nx. The underlying mechanism involves suppression of intrarenal RAS as evidenced by reduced renal renin activity, AGT, and Ang II without affecting the circulating levels of the RAS components. Additionally, we demonstrated that PRO20 treatment prevented renal Wnt/β‐catenin signaling activation, a pathway known to play a key role in pathogenesis of CKD.

PRR has emerged as a key player in pathogenesis of CKD and hypertension through the activation of intrarenal RAS. The renoprotective property of PRO20 has been demonstrated in several experimental models of CKD and hypertension and holds great translational potential in treatment of these devastating diseases (Fang et al., 2018; Luo et al., 2020; Wang et al., 2015; Xu, Lu, Lu, et al., 2017). For example, we have recently demonstrated that PRO20 via inhibiting intrarenal RAS activation attenuated overt proteinuria induced by albumin overload (Fang et al., 2018). However, most of the CKD models used in the previous studies for testing renoprotective action of PRO20 carried a number of limitations. In particular, most of these models don't typically develop features such as end‐stage renal disease (ESRD), as seen in patients with this disease. The 5/6Nx model is perhaps among the best defined models of human ESRD and thus will be well suited for testing renoprotective action of PRO20. Indeed, the 5/6Nx model developed severe impairment of renal function as reflected by ~three‐fold increase in plasma creatinine, a reliable index of renal function in both human and animals. In contrast, PRO20 treatment remarkably, attenuated the rise of plasma creatinine. Similar results were obtained from measurement of BUN. In parallel, improvement was seen in renal pathologies, inflammation, oxidative stress, and fibrosis. Regardless of the underlying mechanism, the present study provides compelling evidence supporting therapeutic potential of this agent in the 5/6Nx model. This is a timely information since there is unmet need for the development of novel renoprotective agents for treatment of CKD. RAS inhibitors such as ACE inhibitors or AT1R antagonists are widely prescribed for patients with CKD but their efficacy is quite limited in that they are unable to prevent the progression of CKD to ESRD. Recent clinical trials have shown promise of sodium‐glucose cotransporter 2 (SGLT2) inhibitors in management of CKD associated with or without type 2 diabetes (Kelly et al., 2019; Nespoux & Vallon, 2020). However, knowledge gap exists in the mechanism of renoprotective action of SGLT2 inhibitors.

The 5/6Nx model is known to be associated with activation of the RAS, especially the intrarenal RAS, as well as inflammation among other mechanisms. In the current study, we found that PRO20 attenuated indices of intrarenal RAS without affecting those of systemic RAS. We also observed elevated TBARS concentrations in the plasma and urine of 5/6Nx model and PRO20 was able to block the elevation of the TBARS. Our data are consistent with previous reports that renal PRR is associated with increased oxidative stress in CKD (Lu et al., 2016). Here, we also found that PRO20 could inhibit over‐expression of inflammation factors, such as IL‐1β, IL‐6, IL‐8, and MCP‐1 in renal cortex in 5/6Nx rats. We also found that PRO20 significantly reduced fibrotic factor FN, α‐SMA, COL‐1, TGF‐β1, which was increased by 5/6Nx. These results suggested that 5/6Nx‐induced upregulation of TBARS, FN, α‐SMA, COL‐1, TGF‐β1, IL‐1β, IL‐6, IL‐8, and MCP‐1 was mediated through PRR. Actually, PRR plays a multitude functions in a RAS‐dependent and independent manner (Ichihara & Yatabe, 2019). PRR can promote kidney injury and fibrosis by amplifying Wnt/β‐catenin signaling (Li, Zhou, et al., 2017). These findings suggest PRR can act in RAS‐independent manners in the pathogenesis of CKD. Our findings are in line with recent findings showing that PRR is required for Wnt/β‐catenin signaling activation (Li, Zhou, et al., 2017).

AGT produced in the liver and filtered through the glomerular basement membrane is the primary source of intrarenal Ang Ⅱ (Matsusaka et al., 2012). Renal proximal tubules also generates AGT (Kobori et al., 2007). In our present study, immunostaining showed that PRR labeling was mostly restricted to the intra‐glomerular area and was enhanced by 5/6Nx. We also found that urinary AGT and Ang Ⅱ were reduced by PRO20. It is possible that AGT excreted from the glomerulus was reduced by PRO20 and led to decreased Ang Ⅱ generation. Whether PRO20 influences the glomerular permeability of AGT, or the generation of AGT in the proximal tubules, or both, is an interesting topic that warrants further pursuit.

A large number of clinical studies have shown that sPRR increases in patients’ circulation under various physiological and pathological conditions, suggesting its value as a biomarker of disease (Hase et al., 2017; Morimoto et al., 2014; Yamashita et al., 2019). In the present study, we also found urinary sPRR expression increased greatly in 5/6Nx rats, and PRO20 decreased sPRR expression. Further research effort is still needed to determine whether sPRR is a marker of early renal injury in proteinuria nephropathy. An intriguing possibility is that sPRR may not only serve as a predictive biomarker but also play an active role in pathogenesis of CKD. In a recent study, our group found that, albumin‐induced inflammatory signaling pathways was mediated by the activation of sPRR in renal tubular cells, which provides a rationale for conducting the current *in vivo* investigation (Fang et al., 2017). Exposure of these cells to albumin overload induced accumulation of sPRR secretion to at least 10‐fold more than the control; this tendency was in congruity with renal sPRR abundance and urinary sPRR excretion in albumin overload rats. Moreover, we found site‐1 protease (S1P) rather than furin or ADAM19 as the main source of albumin overload‐induced sPRR excretion (Fang et al., 2017). This finding is consistent with the study by Nakagawa et al. (Nakagawa et al., 2017), who found that similarly report S1P is required for the cleavage of PRR. Future research is needed to examine the possible effect of proteinuria on S1P activity/expression and the pathogenesis of proteinuria‐induced nephropathy *in vivo* still remains to be fully elucidated.

Taken together, the present results suggest that the PRR mediates renal injuries through activation of the intrarenal RAS and Wnt/β‐catenin signaling in an animal model with 5/6Nx nephropathy. It seems reasonable to conclude that PRR is not only to initiate activation of the intrarenal local RAS, but it also can promote kidney destruction by amplifying Wnt/β‐catenin signaling. Furthermore, blockade of the PRR activation by PRO20 obviously inhibited PRR‐mediated β‐catenin activation and its downstream FN and α‐SMA expression, suggesting PRR‐mediated Wnt/β‐catenin signaling. It is interesting to note that PRR‐mediated Wnt/β‐catenin signaling activation that requires renin/prorenin engagement. These findings may disagree with the *in vitro* observation that PRR augments Wnt/β‐catenin signaling by a mechanism that is independent of renin and prorenin (Li, Zhou, et al., 2017). The reason for such discrepancy is unclear but may be related to differences in the use of *in vivo* versus *in vitro* experimental approaches.

In summary, the present study is the first to report the therapeutic potential of a PRR decoy inhibitor PRO20 in a rat model of 5/6Nx. PRO20 exhibits robust renoprotective action in improving 5/6Nx‐induced renal failure, inflammation, oxidative stress, and renal fibrosis. The underlying mechanism involves suppression of intrarenal RAS and β‐catenin signaling. These results call for clinical elevation of this agent in patients with CKD and hypertension.

## CONFLICT OF INTEREST

None.

## AUTHOR CONTRIBUTIONS

HF conceived the project. HF and YW designed the project. YW, KX, FG, HW, JZ, and HF preformed experiments and analyzed data. HF, YW, CL, and TY wrote the paper. HF supervised the project.
